# Romosozumab versus bisphosphonates for preventing subsequent vertebral fractures after balloon kyphoplasty: comparison using data from two prospective multicenter studies

**DOI:** 10.1093/jbmrpl/ziae137

**Published:** 2024-10-29

**Authors:** Hiroyuki Inose, Shinji Takahashi, Masatoshi Teraguchi, Tsuyoshi Kato, Kentaro Yamada, Hiroyuki Yasuda, Masaki Terakawa, Masakazu Minetama, Masaki Tomori, Yukihiro Nakagawa, Toshitaka Yoshii

**Affiliations:** Department of Orthopaedic Surgery, Dokkyo Medical University Saitama Medical Center, Saitama 343-8555, Japan; Department of Orthopedics, Institute of Science Tokyo, Tokyo 113-8519, Japan; Department of Orthopaedic Surgery, Osaka Metropolitan University Graduate School of Medicine, Osaka 545-8585, Japan; Department of Orthopedic Surgery, Wakayama Medical University, Wakayama 641-8510, Japan; Department of Orthopaedics, Ome Municipal General Hospital, Tokyo 198-0042, Japan; Department of Orthopedics, Institute of Science Tokyo, Tokyo 113-8519, Japan; Department of Orthopaedic Surgery, Osaka General Hospital of West Japan Railway Company, Osaka 545-0053, Japan; Department of Orthopaedic Surgery, Osaka General Hospital of West Japan Railway Company, Osaka 545-0053, Japan; Spine Care Center, Wakayama Medical University Kihoku Hospital, Wakayama 649-7113, Japan; Department of Orthopedic Surgery, Saiseikai Kawaguchi General Hospital, Saitama 332-8558, Japan; Spine Care Center, Wakayama Medical University Kihoku Hospital, Wakayama 649-7113, Japan; Department of Orthopedics, Institute of Science Tokyo, Tokyo 113-8519, Japan

**Keywords:** osteoporotic vertebral fracture, balloon kyphoplasty, osteoporosis, romosozumab, bisphosphonate

## Abstract

Preventing subsequent fractures after vertebral augmentation is a critical clinical concern. The purpose of this study was to compare the effect of romosozumab and bisphosphonate administration on the occurrence of subsequent vertebral fractures after balloon kyphoplasty (BKP) and to identify factors associated with the occurrence of subsequent vertebral fractures. The study compared 24 patients who underwent BKP and received romosozumab with 58 control patients who underwent BKP and received bisphosphonates, all within 2 months of acute osteoporotic vertebral fracture and showing unfavorable magnetic resonance imaging prognostic factors. The primary outcome was the occurrence of subsequent fracture, and the secondary outcomes were improvement in back pain visual analog scale (VAS) score. Furthermore, logistic regression analysis was conducted to adjust for confounding factors and assess the effect of osteoporosis treatment type on subsequent vertebral fractures following BKP. Subsequent vertebral fractures occurred in 16 patients in the bisphosphonate group and in 1 patient in the romosozumab group (*p* = .02). There were no significant differences between the 2 groups in VAS scores and their change from preoperatively to 6 months after surgery. The multivariable logistic regression analysis identified the type of osteoporosis treatment as an independent factor associated with the occurrence of subsequent vertebral fractures (Odds ratio, 18.30, *p* = .02). This prospective, multicenter study demonstrates that romosozumab is more effective than bisphosphonates in preventing subsequent vertebral fractures within 6 months after BKP. Romosozumab’s superior efficacy in reducing subsequent vertebral fractures may lead to improved long-term outcomes and quality of life, potentially making it a preferred treatment option over bisphosphonates for patients undergoing BKP.

## Introduction

The number of patients suffering from vertebral fractures is increasing with the aging of society.^1^ While good postoperative outcomes of vertebral augmentation (vertebroplasty and balloon kyphoplasty [BKP]) have been reported,^2^^,^^3^ there is a high rate of postoperative subsequent vertebral fractures after vertebral augmentation.^4^^,^^5^ The incidence of adjacent vertebral fractures after vertebral augmentation varies, ranging from 12% to 52%.^6^ In addition, patients with vertebral fractures are osteoporotic, which may lead to subsequent vertebral fractures in areas other than the adjacent vertebrae.^7^ Of note, vertebral fractures are associated with a reduced quality of life and, furthermore, the risk of further fractures increases with the number of vertebral fractures.^8^ Accordingly, the prevention of subsequent vertebral fractures following vertebral augmentation is a critical clinical concern.

Recent advances in molecular biology have elucidated bone remodeling mechanisms, leading to the development of various drugs for osteoporosis.^9^ Bisphosphonates, which inhibit bone resorption, have been the most widely used drugs for the treatment of osteoporosis, but recently romosozumab, which promotes bone formation and inhibits bone resorption, has enabled rapid increases in bone mass and strength in a short time.^10^^,^^11^ Indeed, a finite element analysis study showed that romosozumab strongly increased integral, cortical, and trabecular bone strength over a 6-month period compared with alendronate.^12^ We therefore hypothesized that the use of romosozumab in the perioperative period of BKP would result in fewer subsequent fractures than with bisphosphonates, which has been the standard of care. The purpose of this study was to compare the effect of romosozumab and bisphosphonate administration on the occurrence of subsequent vertebral fractures after BKP and to identify factors associated with the occurrence of subsequent vertebral fractures.

## Materials and methods

### Patients

We undertook a multicenter prospective intervention study at 7 institutions in Japan between January 2021 and March 2024. Patients older than 65 years were eligible for enrollment if they had an acute osteoporotic vertebral fracture with estimated fracture age 2 months or less and a high-intensity (fluid) or diffuse low-intensity area in fractured vertebrae on T2 weighted magnetic resonance imaging (MRI).^13^ These MRI findings were associated with nonunion, reduced activities of daily living, and chronic back pain.^13^^,^^14^ MRI was assessed in each institution. On the initial visit, a new vertebral fracture was diagnosed if the following were present: acute back pain, a deformed vertebral body on radiographs, and abnormal intensity within the vertebral bodies on MRI. These fractures had low-energy injury mechanisms (ie, spontaneous fractures, fall from standing, etc.). Exclusion criteria included abnormal renal function, neurological symptoms, pathological fractures, dementia, and patients not meeting the criteria for romosozumab administration (eg, patients with a history of cardiovascular events). Enrolled participants had a back pain score of 4 points or more on a 0 to 10 scale. This study was performed in line with the principles of the Declaration of Helsinki. The protocol and consent form were approved by local ethics committees (M2020-278). Participants gave written informed consent before enrollment.

Overall, 68 patients met the inclusion criteria, with 42 subsequently excluded. The reasons for exclusion encompassed 5 patients with abnormal renal function, 2 patients with neurological symptoms, 7 patients with pathological fractures, 6 patients with dementia, 7 patients who did not meet the criteria for romosozumab administration, and 15 patients who declined to participate in the study (primarily due to concerns about frequent outpatient visits during the COVID-19 pandemic and financial considerations). The remaining 26 patients were enrolled in the study, initiated treatment with romosozumab, and subsequently underwent BKP. Of these, 2 were lost to follow-up. As a result, 24 patients completed the 6-month follow-up. At the time of enrollment, patients underwent plain radiographs, computed tomography (CT), and MRI of the spine. Six months after BKP, patients underwent follow-up radiographs and CT scans to evaluate the potential occurrence of subsequent vertebral fractures.

Kyphoplasty was performed with introducer instruments, inflatable bone tamps, and polymethylmethacrylate bone cement and delivery devices (Medtronic Spine LLC, Sunnyvale, CA, or Depuy Synthes, Bettlach, Switzerland), using a percutaneous, bilateral, and transpedicular approach. All procedures were done under general anesthesia. All participants received analgesics, back braces, physiotherapy, rehabilitation programs, and walking aids according to standard practices of participating physicians and hospitals.

For the control group, we selected 58 patients with acute osteoporotic vertebral fractures from a previously reported multicenter prospective cohort study conducted at 10 institutions in the Osaka area of Japan between April 2015 and September 2017.^15^ These patients underwent BKP, exhibited high-intensity or diffuse low-intensity areas in fractured vertebrae on T2-weighted MRI, and began treatment with bisphosphonates preoperatively. The cohort study consisted of 106 patients older than 65 years who had acute osteoporotic vertebral fractures with estimated fracture age 2 months or less. At the time of enrollment, patients underwent plain radiographs and MRI of the spine. Six months after BKP, patients underwent follow-up radiographs and MRI. Physicians referred participants for treatment with vitamin D, antiresorptive agents, or anabolic agents. Of these, 58 patients who received bisphosphonates were selected to serve as historical controls for this study.

### Outcomes

The primary outcome was a reduction in subsequent fractures after BKP between romosozumab and bisphosphonate groups. In this study, vertebral fracture was defined as grade 2 or higher according to the semiquantitative method, ie, a vertebral fracture in this study was defined as a reduction in anterior vertebral height of 25% or more.^16^

The secondary outcomes were the difference in change from baseline to 6 months in the back pain visual analog scale (VAS) scores.

### Statistical analysis

We performed paired t-test to identify differences in the VAS scores between before and after 6 months of romosozumab treatment. We then performed comparisons between the romosozumab and bisphosphonate groups. We used Fisher exact test or chi square test to compare nominal variables, and Mann-Whitney U test was used to analyze continuous variables. In addition, the VAS scores at preoperative and 6 months after BKP were compared in the groups with and without subsequent vertebral fractures, respectively. We then conducted a multivariable logistic regression analysis to examine the effect of osteoporosis treatment type on subsequent vertebral fractures following BKP, adjusting for potential confounding factors. To adjust for possible confounding, the following variables were included in the model: age, sex, bone density, prior osteoporosis treatment history, the presence of preexisting vertebral fractures,^17^^,^^18^ and fracture lesion (thoracic or thoracic-lumbar junction fractures).^18^ Test results were considered significant at *p* < .05. All *p* values were 2-sided. All analyses were performed using JMP version 14 software (SAS Institute, Cary, NC).

Sample size calculations for romosozumab group assumed a difference of 75% in rate of reduction in the occurrence of subsequent vertebral fractures after BKP (ie, 40 % in bisphosphonate group, 10 % in romosozumab group). With an alpha of 0.05, we would achieve 80% power to detect a difference with 32 patients per arm. However, enrollment was slower than expected, possibly due to the impact of the COVID-19 outbreak, so the final enrollment in the romosozumab group was 26 patients as of the planned study completion date.

## Results

### Patient characteristics and clinical outcomes

A total of 82 patients were included in the study. The baseline characteristics of each group are shown in Table 1. The romosozumab group was older than the bisphosphonate group (*p* = .006), but no differences were observed in other factors.

**Table 1:** Baseline patient characteristics of bisphosphonate and romosozumab groups.

	Bisphosphonate (*n* = 58)	Romosozumab (*n* = 24)	*p*-value
**Age (yr)**	78.6 ± 5.5	82.1 ± 5.1	0.006^a^
**Sex, n (%)**	Female 48 (83) Male 10 (17)	Female 19 (79) Male 5 (21)	0.70
**BMI**	22.1 ± 3.1	22.4 ± 4.6	>0.99
**Bone density** **YAM**	70.4 ± 11.2	73.1 ± 14.0	0.49
**Mean number of preexisting vertebral fracture per patient**	0.8 ± 1.2	1.3 ± 1.6	0.33
**Number of patients with preexisting vertebral fracture**	26	13	0.48

a *p* < 0.05

Abbreviations: BMI, body mass index; YAM, young adult mean

The clinical outcomes of the study are presented in Table 2. Subsequent vertebral fractures occurred in 16 patients (27.6%) in the bisphosphonate group and in 1 patient (4.2%) in the romosozumab group (*p* = .02). In addition, the number of subsequent vertebral fractures was 20 in the bisphosphonate group and 1 in the romosozumab group, meaning that the average number of subsequent vertebral fractures occurring after surgery was also higher in the bisphosphonate group than in the romosozumab group (*p* = .02). Low back pain VAS scores showed improvement at the final follow-up compared with preoperative results (7.3 ± 2.8 versus 3.0 ± 2.8, *p* < .001). There were no significant differences between the 2 groups in VAS scores and their change from preoperatively to 6 months after surgery. Groups romosozumab and bisphosphonate were then combined and compared by dividing the entire patient population into 2 groups based on the presence or absence of a subsequent vertebral fracture after BKP. The VAS scores at 6 months postoperatively tended to be higher in the fracture group than in the no-fracture group (3.9 ± 3.3 versus 2.8 ± 2.6, *p* = .18).

**Table 2:** Comparison of clinical outcomes.

	Bisphosphonate (*n* = 58)	Romosozumab (*n* = 24)	*p*-value
**Mean number of subsequent vertebral fractures**	0.4 ± 0.7	0.04 ± 0.2	0.02^a^
**Total number of subsequent vertebral fracture**	20	1	0.02^a^
**Number of patients with subsequent vertebral fractures, n (%)**	16 (27.6)	1 (4.2)	0.02^a^
**Preoperative back pain VAS score**	7.3 ± 2.8	7.2 ± 3.1	0.93
**Postoperative 6 months back pain VAS score**	3.0 ± 2.6	3.1 ± 3.2	0.75
**Changes in VAS**	4.3 ± 3.7	4.1 ± 2.9	0.45

a *p* < 0.05

VAS, visual analog scale

### Factors associated with subsequent vertebral fractures

We performed univariate logistic regression analysis to identify factors that associated with subsequent vertebral fractures. As a result, the type of osteoporosis treatment was associated with the occurrence of subsequent fractures (Odds ratio 8.76, *p* = .04) (Table 3). We then performed multivariable logistic regression analyses to assess the effect of osteoporosis treatment on the occurrence of subsequent vertebral fractures after BKP, adjusting for potential confounding factors. Age, sex, bone density, prior osteoporosis treatment history, the presence of preexisting vertebral fractures, and fracture lesion were selected as potential confounding factors. Multivariable logistic regression analysis revealed that the type of osteoporosis treatment was an independent risk factor for the occurrence of subsequent vertebral fractures after BKP (Odds ratio 18.30, *p* = .02) (Table 4).

**Table 3:** Factors associated with subsequent vertebral fractures. Univariate logistic regression analysis.

	Odds ratio	95% Confidence interval	*p*-value
**Age**	0.94	0.85-1.04	0.24
**Sex (female/male)**	0.66	0.18-2.42	0.53
**BMI**	0.90	0.76-1.06	0.20
**Bone density** **YAM**	1.04	0.99-1.09	0.15
**Number of preexisting vertebral fracture**	1.11	0.76-1.64	0.59
**Presence of preexisting vertebral fracture**	2.36	0.78-7.16	0.13
**Prior osteoporosis treatment history**	1.28	0.43-3.80	0.66
**Type of osteoporosis treatment (bisphosphonate/romosozumab)**	8.76	1.09-70.36	0.04^a^
**Fracture lesion (thoracic or thoracic-lumbar junction)**	3.26	0.39-27.20	0.28
**Preoperative back pain VAS score**	1.00	0.98-1.02	0.92

a *p* < 0.05

BMI, body mass index; YAM, young adult mean; VAS, visual analog scale

**Table 4:** Multivariable logistic regression analysis of the association between the type of osteoporosis treatment and the occurrence of subsequent vertebral fractures.

	Odds ratio	95% Confidence interval	*p*-value
**Type of osteoporosis treatment (bisphosphonate/romosozumab)**	18.30	1.62-207.22	0.02^a^
**Presence of preexisting vertebral fracture**	5.58	1.27-24.43	0.02^a^
**Bone density YAM**	1.07	0.998-1.15	0.06
**Prior osteoporosis treatment history**	2.10	0.47-9.47	0.33
**Sex (female/male)**	0.43	0.06-2.93	0.39
**Fracture lesion (thoracic or thoracic-lumbar junction)**	2.26	0.20-25.61	0.49
**Age**	1.00	0.87-1.16	0.98

a *p* < 0.05

YAM, young adult mean

## Discussion

In this study, patients receiving romosozumab had significantly fewer subsequent vertebral fractures than patients receiving bisphosphonates. These findings are consistent with a recently published retrospective study showing that romosozumab reduced subsequent fractures more than bisphosphonates.^19^ However, our study differs in that it was a prospective, multicenter study and defined subsequent vertebral fractures after BKP as SQ grade 2 or greater. Romosozumab was shown in a Phase 2 study to increase bone mineral density more than bisphosphonates at 3 months after administration,^20^ and this bone mineral density enhancing effect may have helped reduce fractures in this study. Therefore, in order to prevent the occurrence of severe vertebral fractures, it may be meaningful to administer romosozumab during the perioperative period of vertebroplasty.

In this study, BKP and osteoporosis treatment reduced VAS values by approximately 4 points compared with preoperative levels, which is significantly higher than the minimal clinically important difference of 1.2 points for back pain VAS score.^21^ Although the indication for vertebroplasty in the treatment of acute osteoporotic vertebral fractures remains controversial,^22^^,^^23^ BKP for patients meeting the inclusion criteria of this study is considered effective. A more rigorous validation of the effectiveness of this treatment strategy would require a randomized controlled trial (RCT) in this patient group comparing the outcomes of sham surgery and BKP.

The results of the VAS scores were the same as those in the study by Ueno et al.^19^, with no change between the bisphosphonate and romosozumab groups. However, when comparing groups with and without subsequent fractures, the VAS scores at the final follow-up tended to be higher in the group with fractures in this study. The occurrence of subsequent vertebral fractures after vertebral fractures has been reported to lead to residual back pain.^24^ In a multicenter prospective study, a group of patients who developed a subsequent vertebral fracture after an osteoporotic vertebral fracture reported more back pain than a group of patients who did not develop a subsequent vertebral fracture.^25^ Therefore, the absence of a statistically significant difference in VAS scores for low back pain between the subsequent fracture group and the no subsequent fracture group, rather than between drug treatments, may be attributable to insufficient statistical power due to limited sample size. However, even if there were a difference in the incidence of subsequent vertebral fractures between the romosozumab and bisphosphonate groups, a comparison of pain between these groups would inherently include patients who did not develop subsequent vertebral fractures. Consequently, it may be challenging to detect a statistically significant difference in VAS scores between the romosozumab and bisphosphonate groups. Since multiple vertebral fractures increase the probability of new vertebral fractures,^8^ the prevention of subsequent fractures is of paramount importance.

Logistic regression analysis showed that the type of osteoporosis treatment was associated with the occurrence of subsequent vertebral fractures. In previous studies, subsequent vertebral fractures occur early after BKP and vertebroplasty.^19^^,^^26^ Therefore, even though romosozumab is a potent bone-anabolic agent, its effect in preventing subsequent vertebral fractures has been considered limited. Although this study did not distinguish between adjacent vertebral fractures and vertebral fractures at other sites due to the sample size, the results of this study demonstrated that administration of romosozumab can reduce the likelihood of developing severe vertebral fractures of SQ grade 2 or higher within 6 months after BKP. A biomechanical study has shown that treatment with romosozumab significantly increases vertebral strength as early as 3 months after administration.^27^ Therefore, even if an early postoperative vertebral body injury occurs, it may result in only a minor deformity in patients undergoing treatment with romosozumab. Furthermore, this study showed that the type of osteoporosis treatment has a stronger impact on the occurrence of subsequent fractures than bone density or the number of preexisting vertebral fractures. Therefore, preoperative and perioperative romosozumab administration is recommended over bisphosphonate if one wishes to reduce the occurrence of subsequent severe vertebral fractures in patients scheduled for BKP, regardless of bone density or the presence of preexisting vertebral fractures.

This study has several limitations. First, it is not an RCT that allows a more precise comparison and analysis of the effects of romosozumab and bisphosphonates on the occurrence of subsequent vertebral fractures after BKP surgery. However, at a time when anabolic agents are recommended for severe osteoporosis, we do not believe it is ethical to conduct an RCT assigning patients who are eligible for these treatments to bisphosphonate treatment. Therefore, we conducted a prospective single-arm intervention study with a historical control group. Second, the follow-up period is as short as 6 months to be identical to the historical control. However, most subsequent fractures after kyphoplasty occur within 60 days.^26^ In fact, Ueno et al.^19^ reported that only 9% of fractures occurred after 6 months during a 1-year period. Therefore, we believe that our study period of 6 months was sufficient to detect the fractures necessary for analysis. However, further research, including RCTs with larger sample sizes, is needed to confirm these findings and establish optimal treatment protocols for this patient population.

## Conclusions

This prospective, multicenter study demonstrates that romosozumab is more effective than bisphosphonates in preventing severe vertebral fractures (SQ grade 2 or higher) within 6 months after BKP. While both treatments improved pain scores, romosozumab’s superior efficacy in reducing subsequent vertebral fractures may lead to improved long-term outcomes and quality of life, potentially making it a preferred treatment option over bisphosphonates for patients undergoing BKP.

## Consent to participate

Participants gave written informed consent before enrollment.

## Data Availability

The data that support the findings of this study are available from the corresponding author, upon reasonable request.
